# LEDGINs, a novel class of antivirals targeting HIV integrase during integration and assembly

**DOI:** 10.1186/1742-4690-10-S1-O44

**Published:** 2013-09-19

**Authors:** Zeger Debyser

**Affiliations:** 1 Molecular Virology and Gene Therapy KU Leuven, Flanders, Belgium

## 

Integrase strand transfer inhibitors (INSTIs) such as raltegravir have become a key component of antiviral therapy. We have recently described the development of a novel class of integration inhibitors, 2-(quinolin-3-yl)acetic acid derivatives, that potently block HIV replication (Christ *et al. Nature Chemical Biology* 2010). Unlike clinically approved INSTIs, these compounds do not bind to the catalytic site of HIV integrase (IN). As allosteric inhibitors they bind to the LEDGF/ p75 binding pocket in integrase, hence the class name LEDGINs. This prevents the interaction with LEDGF/p75, a molecular tether of HIV IN, and inhibits the catalytic activities of IN. Detailed mechanism of action studies reveal that the allosteric mode of inhibition is due to the stabilization of the IN dimer. Recently, we demonstrated that LEDGINs also inhibit late stage HIV replication by stabilization of IN multimers. Evidence suggests that LEDGF/p75 may play a role in this process. Lack of cross-resistance with available IN inhibitors and potency due to the multistep inhibition, support the further clinical development of LEDGINs.

